# Smart Sensing Technologies for Personalised e-Coaching

**DOI:** 10.3390/s18061751

**Published:** 2018-05-29

**Authors:** Oresti Banos, Hermie Hermens, Christopher Nugent, Hector Pomares

**Affiliations:** 1 Biomedical Signals and Systems Group, University of Twente, 7522 NB Enschede, The Netherlands; H.Hermens@rrd.nl; 2 CITIC-UGR, University of Granada, E-18015 Granada, Spain; hector@ugr.es; 3 Telemedicine Group, Roessingh Research and Development, 7500 AH Enschede, The Netherlands; 4 Smart Environments Research Group, Ulster University, Newtownabbey BT37 0QB, UK; cd.nugent@ulster.ac.uk

**Keywords:** e-coaching, wearable sensors, smartphones, smart objects, activity recognition, context-awareness, behavior change

## Abstract

People living in both developed and developing countries face serious health challenges related to sedentary lifestyles. It is therefore essential to find new ways to improve health so that people can live longer and age well. With an ever-growing number of smart sensing systems developed and deployed across the globe, experts are primed to help coach people to have healthier behaviors. The increasing accountability associated with app- and device-based behavior tracking not only provides timely and personalized information and support, but also gives us an incentive to set goals and do more. This paper outlines some of the recent efforts made towards automatic and autonomous identification and coaching of troublesome behaviors to procure lasting, beneficial behavioral changes.

## 1. Introduction

Lifestyle choices can have a tremendous impact on people’s health and wellness. Avoiding unhealthy habits is nowadays a priority, and to achieve this goal, ground-breaking mechanisms are required to automatically and autonomously identify and eventually change people’s behaviors. An increasing number of smart, ubiquitous sensing technologies are being developed all over the world to coach people on healthier and more responsible behavior, providing them with timely personalized information and support. This Special Issue aims at bringing together the latest experiences, findings, and developments on smart sensing, modeling, and understanding of human behavior for the provision of personalized coaching and support services.

## 2. Contributions

This special issue has collected eleven outstanding papers touching upon different aspects of smart sensing for e-coaching applications. In the following, a brief summary of the scope and main contributions of each of these papers is provided as a teaser for the interested reader.

As it has been pointed out in the introduction of this editorial, sedentary lifestyles are one of the major causes of health problems in our society. In “Personalized Physical Activity Coaching: A Machine Learning Approach” [1], the authors report their experiences with different machine learning algorithms to timely estimate the probability for a given subject of achieving a personalized step goal. They integrated this model into a web app that helps the expert predict, with a level of certainty, whether the subject will be able to achieve their goal based on their most immediate prior performance.

Not only is it important to detect or predict the activity patterns of a given user, but also to ensure that the users are properly informed and to encourage people to change at the point of need. In “Active2Gether: A Personalized m-Health Intervention to Encourage Physical Activity” [2], the authors present a system that monitors young adults’ physical behavior by using wearable and mobile sensors, facilitates social comparison, and provides personalized intelligent feedback. The authors share interesting lessons learned during the design, implementation, and evaluation of the system, such as the difficulties encountered while directly accessing the data of commercially available activity trackers.

Promoting or encouraging people to exercise is normally positive. In some cases, like sports, it is also important to provide tailored instructions to avoid possible injuries. In “The Feasibility and Usability of RunningCoach: A Remote Coaching System for Long-Distance Runners” [3], the authors propose a mobile system that monitors running cadence levels, which are shown to be strongly associated with running-related injuries. The system uses this information to estimate the optimal cadence for the runner, which can in turn modulate their running style while reducing the risk of injuries.

Minimizing risks is of utmost importance to ensure a plentiful life, especially when it comes to more serious heart-related aspects. In “Real-Time Monitoring in Home-Based Cardiac Rehabilitation Using Wrist-Worn Heart Rate Devices” [4], the authors present a system that facilitates the continuous monitoring of heart-rate activity during home-based rehabilitation sessions. The approach leverages existing clinical guidelines for monitoring the heart rate of the patient. The registered data is then modeled through fuzzy techniques in order to realize remote cardiac rehabilitation sessions.

The recognition of physical activities has been widely explored during recent years. However, not much effort has been put into the detection of rather complex activities such as eating. In “Modular Bayesian Networks with Low-Power Wearable Sensors for Recognizing Eating Activities” [5], the authors propose an approach combining wearable and mobile sensors to detect such complex activity. The approach builds on a probabilistic Bayesian network with a modular and tree-structured form to reduce time complexity and increase scalability. Their results show that good detections can be made even when some of the sensor values have a very heterogeneous pattern or are missing.

Recognizing eating patterns turns to be quite useful when trying to understand dietary habits. Thus, it is necessary to provide recommendations and feedback to users, to support them in improving their routines. In “Smart Device-Based Notifications to Promote Healthy Behavior Related to Childhood Obesity and Overweight” [6], the authors present a prototype targeted at parents to increase their awareness towards nutrition and exercise. The system is based on smart objects that can be attached to the fridge to remind parents visually to prepare a healthy snack or to bring the necessary sports equipment. According to the authors, the system has been rated positively by both nutritionists and physical therapists.

The popularity of the internet-of-things is making smart objects quite an interesting technology for e-coaching. In “Creating Affording Situations: Coaching through Animate Objects” [7], the authors explore the use of these devices to cue actions in everyday activities. Patients with neurological disorders can often struggle with such activities, for example, by confusing the sequence in which tasks should be performed or forgetting which action can be realized using a given object. This work presents a set of prototypes that exploit the use of lights and audio on animated objects to influence activity by reducing uncertainty, which in turn can challenge pre-learned action sequences.

Smart objects are typically confined to indoor environments. Understanding how people behave in such environments is—next to other reasons such as energy management, security, and safety—of much relevance. In “Location-Enhanced Activity Recognition in Indoor Environments Using Off the Shelf Smart Watch Technology and BLE Beacons” [8], the authors propose a system that uses commercial off-the-shelf smartwatches and Bluetooth low energy beacons. By combining these two technologies, the authors show clear improvements in the recognition of activities with respect to prior works that exclusively rely on either device.

Detecting the activity a user is performing is as important as understanding the context around such activity. In “Context Mining of Sedentary Behaviour for Promoting Self-Awareness Using a Smartphone” [9], the authors exploit sensors embedded into regular smartphones to mine the temporal context of passive behaviors. The proposed system uses the inertial sensors to first differentiate between active or still; if the person is categorized into the latter, then environmental audio is captured and processed to identify the specific context. This information is then used to trigger a coaching action to interrupt such a sedentary behavior and stimulate an active one.

People often attribute their sedentariness to being extremely occupied over the day. Thus, finding ways to introduce exercise into their lives has become of much relevance. In “Increasing the Intensity over Time of an Electric-Assist Bike Based on the User and Route: The Bike Becomes the Gym” [10], the authors present an application that increases the pedaling intensity for an electric pedal-assist-bike. The system personalizes the “extra effort” a person has to put into their normal cycling based on the user’s strength and the route’s characteristics. A social component also motivates interaction and competition between users, based on a scoring system that shows the level of their performances.

There is a great realm of solutions for e-coaching building on smart technologies as can be seen from the examples outlined above. However, as coaching on healthy behaviors is a broad challenge, not only is it important to develop individual solutions but also infrastructures that can accommodate and combine several of them. In “Design and Evaluation of a Pervasive Coaching and Gamification Platform for Young Diabetes Patients” [11], the authors describe a platform that integrates mobile digital coaching systems connected with wearable sensors, serious games, and patient web portals to personal health records, with the aim to support patients with chronic conditions and their caregivers in realizing the ideality of self-management. This work shows how behavioral change theories can be engrained in the design of e-coaching technologies, with the aim of developing successful and long-lasting interventions in healthcare.
